# Kinetics and 28-day test–retest repeatability and reproducibility of [^11^C]UCB-J PET brain imaging

**DOI:** 10.1177/0271678X20964248

**Published:** 2020-10-08

**Authors:** Hayel Tuncel, Ronald Boellaard, Emma M Coomans, Erik FJ de Vries, Andor WJM Glaudemans, Paula Kopschina Feltes, David V García, Sander CJ Verfaillie, Emma E Wolters, Steven P Sweeney, J Michael Ryan, Magnus Ivarsson, Berkley A Lynch, Patrick Schober, Philip Scheltens, Robert C Schuit, Albert D Windhorst, Peter P De Deyn, Bart NM van Berckel, Sandeep SV Golla

**Affiliations:** 1 Department of Radiology & Nuclear Medicine, Amsterdam Neuroscience, Amsterdam UMC, Amsterdam, The Netherlands; 2 Department of Nuclear Medicine and Molecular Imaging, University Medical Center, University of Groningen, Groningen, The Netherlands; 3 Department of Neurology, Alzheimer Center Amsterdam, Amsterdam Neuroscience, Amsterdam UMC, Amsterdam, The Netherlands; 4 Rodin Therapeutics Inc., Cambridge, MA, USA; 5 Department of Anaesthesiology, Amsterdam UMC, Amsterdam, The Netherlands; 6 Department of Neurology, University of Groningen, University Medical Center Groningen, Groningen, The Netherlands; 7 Alzheimer Research Center, University of Groningen, University Medical Center Groningen, Groningen, The Netherlands

**Keywords:** Alzheimer’s disease, [11C]UCB-J, kinetic modelling, PET, SV2A

## Abstract

[^11^C]UCB-J is a novel radioligand that binds to synaptic vesicle glycoprotein 2A (SV2A). The main objective of this study was to determine the 28-day test–retest repeatability (TRT) of quantitative [^11^C]UCB-J brain positron emission tomography (PET) imaging in Alzheimer’s disease (AD) patients and healthy controls (HCs). Nine HCs and eight AD patients underwent two 60 min dynamic [^11^C]UCB-J PET scans with arterial sampling with an interval of 28 days. The optimal tracer kinetic model was assessed using the Akaike criteria (AIC). Micro-/macro-parameters such as tracer delivery (K_1_) and volume of distribution (V_T_) were estimated using the optimal model. Data were also analysed for simplified reference tissue model (SRTM) with centrum semi-ovale (white matter) as reference region. Based on AIC, both 1T2k_V_B_ and 2T4k_V_B_ described the [^11^C]UCB-J kinetics equally well. Analysis showed that whole-brain grey matter TRT for V_T_, DVR and SRTM BP_ND_ were –2.2% ± 8.5, 0.4% ± 12.0 and –8.0% ± 10.2, averaged over all subjects. [^11^C]UCB-J kinetics can be well described by a 1T2k_V_B_ model, and a 60 min scan duration was sufficient to obtain reliable estimates for both plasma input and reference tissue models. TRT for V_T_, DVR and BP_ND_ was <15% (1SD) averaged over all subjects and indicates adequate quantitative repeatability of [^11^C]UCB-J PET.

## Introduction

Many neurodegenerative and neurological disorders such as Alzheimer’s disease (AD),^1,2^ Parkinson’s disease (PD),^3^ epilepsy^4,5^ and autism spectrum disorder^6^ are associated with synaptic pathology. More specifically, in AD, cognitive impairment is highly correlated with synaptic loss in the association cortex and limbic system.^7,8^ Synaptic disruption is thought to be associated with toxic β-amyloid or tau oligomers and is already observed in the earliest clinical stages of AD.^9^ These findings suggest that the ability to assess synaptic density in vivo could improve clinical research in AD and potentially could serve as a valuable surrogate marker for disease severity in clinical trials.

(R)-1-((3-([^11^C]methyl)pyridin-4-yl)methyl)-4-(3,4,5-trifluorophenyl)pyrrolidin-2-one, also known as [^11^C]UCB-J, is a radioligand with high affinity and specificity for synaptic vesicle glycoprotein 2A (SV2A).^10–12^ [^11^C]UCB-J is a derivative of levetiracetam,^13^ an FDA and EMA approved anti-epileptic drug. SV2A is a member of a small family of synaptic vesicle proteins and is the most widespread isoform present in glutamatergic and GABAergic neurons.^10^ [^11^C]UCB-J binds to SV2a in pre-synaptic terminals and, therefore, could reflect synaptic density. Multiple preclinical animal studies have shown that SV2A and synaptophysin (a widely used presynaptic marker) have essentially a homogeneous distribution across the brain.^10^ Therefore, [^11^C]UCB-J can be used as an imaging agent for brain synaptic pathology in neurological diseases. [^11^C]UCB-J was originally synthesized by Mercier et al.^14^ Nabulsi et al.^12^ have observed high brain uptake of [^11^C]UCB-J in nonhuman primates indicating that [^11^C]UCB-J is a promising radioligand targeting SV2A. The first-in-human [^11^C]UCB-J positron emission tomography (PET) study showed comparable results.^11^

Quantification of specific binding of [^11^C]UCB-J with a validated tracer kinetic model is crucial in clinical studies not only for identification of early synaptic pathology in cross-sectional studies but also for longitudinal assessment of changes in synaptic integrity. A validated kinetic model becomes even more important when it is used as a surrogate marker for assessing the efficacy of disease-modifying drugs. In vivo kinetics for [^11^C]UCB-J have previously been evaluated for HCs and AD patients.^15–18^ For instance, Chen et al.^17^ evaluated the kinetics for [^11^C]UCB-J in AD patients, but no test–retest repeatability (TRT) was reported. Furthermore, Finnema et al.^15^ reported the same-day TRT of UCB-J for 1T2k model; however, only for relatively young HCs. Koole et al.^18^ validated the use of simplified methods such as simplified reference tissue model (SRTM) and standardized uptake value ratios (SUVr) for UCB-J in relatively young HCs only. No TRT was reported for these simplified methods in this study nor was this assessed for AD patients. Moreover, none of these studies addressed the long-term repeatability. The TRT of plasma input models has never been reported for AD patients. The aim of the current study was to assess the long-term (28-day) repeatability of various methods, such as plasma input models, and simplified methods such as SRTM and SUVr in both HCs and AD patients. One of the reasons for the current study design was assessment of a clinical drug intervention study,^19^ where the expected effect size was more than 25% in a 28-day time period. Our current TRT study with a 28-day interval between both scans was designed to closely mimic the condition of the intervention design.^19^ The effect of PET scan duration on the quantification was also evaluated, and regional differences were assessed between HCs and AD patients. The main aims of the current study were: (1) TRT assessment of [^11^C]UCB-J binding to SV2A using kinetic analysis with arterial input function within the 28-day time interval; (2) TRT assessment of V_T_, SRTM BP_ND_ and SUVr in elderly HCs as well as in AD patients.

## Material and methods

### Participants

In this multicentre study, 19 participants were included who all underwent a dynamic [^11^C]-UCB-J PET scan and T1-weighted MRI scan. Two PET scans were excluded due to motion artefacts: one PET scan was not performed due to tracer production failure and one participant discontinued the study after the first PET scan. Eight HCs from the Amsterdam University Medical Center (Amsterdam UMC) and one HC from University Medical Center Groningen (UMCG) were included in the study as well as seven mild-to-moderate AD patients from Amsterdam UMC and one AD patient from UMCG. AD patients were eligible when they had a probable diagnosis of AD defined by National Institute on Aging – Alzheimer’s Association (NIA-AA)^20^ with either abnormal Aβ_42_ in cerebrospinal fluid (CSF) (Aβ_42_ < 813 pg/mL)^21^ or an abnormal amyloid-β PET scan and a Mini-Mental State Examination (MMSE) score between 18 and 26. HCs were recruited through local advertisements. They were eligible for the study if they were cognitively normal without cognitive complaints, absence of significant impairment in cognitive functions or activities of daily living and if the MMSE score was ≥27. The study was conducted in full conformance with the principles of the “Declaration of Helsinki” (as amended in Tokyo, Venice, Hong Kong, Somerset-West, Edinburgh, Washington DC, Tokyo, Seoul and Fortaleza) and was approved by the Medical Review and Ethics Committee (MREC) of Foundation BEBO in Assen, and local feasibility was confirmed by the MREC of Amsterdam UMC and by the MREC of UMCG. Furthermore, all subjects provided written informed consent prior to the study.

### Data acquisition

[^11^C]UCB-J tracer was locally produced at the PET centres of UMCG and Amsterdam UMC according to clinical Good Manufacturing Practice standards. Participants underwent two dynamic PET scans on the same PET–CT system within a given institution (Amsterdam UMC or UMCG) with a 28-day interval. PET scans were acquired on the Ingenuity TF PET/CT scanner (Amsterdam UMC, Philips Medical Systems, Best, The Netherlands) or a Biograph mCT PET-CT scanner (UMCG, Siemens Medical Systems). Prior to the PET scan, a low-dose computed tomography (CT) scan was performed for attenuation correction purposes. After the low-dose CT, a 90 min dynamic PET scan was acquired after a bolus injection of 373 ± 22 MBq [^11^C]UCB-J. Upon interim review (after completion of 9 HC test–retest scans), scan duration was reduced to 60 min for subsequent AD subjects after a bolus injection of 320 ± 39 MBq. During scanning, the head was stabilized to reduce movement artefacts, and subjects were positioned within the centre of axial and transaxial fields of view, such that the orbito-meatal line was parallel to the detectors with the use of laser beams. T1-weighted MRI scans were also acquired for all participants using a 3.0-T Philips Ingenuity Time-of-Flight PET/MR scanner at Amsterdam UMC and using a 1.5-T Siemens Aera at UMCG. At Amsterdam UMC, PET images of 22 frames (1 × 15, 3 × 5, 3 × 10, 4 × 60, 2 × 150, 2 × 300 and 7 × 600 s) or 19 frames (1 × 15, 3 × 5, 3 × 10, 4 × 60, 2 × 150, 2 × 300 and 4 × 600 s) with a matrix size of 128 × 128 × 90 voxels and a final voxel size of 2 × 2 × 2 mm^3^ were reconstructed using 3D row action maximum likelihood algorithm (RAMLA). At UMCG, PET images of 25 frames (1 × 10, 6 × 5, 3 × 10, 4 × 60, 2 × 150, 2 × 300 and 7 × 600 s) or 22 frames (1 × 10, 6 × 5, 3 × 10, 4 × 60, 2 × 150, 2 × 300 and 4 × 600 s) with a matrix size of 400 × 400 × 111 voxels and a final voxel size of 2 × 2 × 2 mm^3^ were reconstructed using 3D ordered-subsets-expectation-maximization (OSEM-TOF) algorithm. Furthermore, all usual corrections for attenuation, scatter, randoms, decay and dead time were performed.

### Blood data acquisition and processing

Before the PET scan, a venous catheter was inserted for injection of the [^11^C]UCB-J solution. In addition, an arterial cannula was inserted in the radial artery to collect blood samples for measurement of the time course of the tracer in plasma, including radioactive metabolite analysis. A maximum of 75 mL arterial blood was sampled continuously over 60 min for HCs and 30 min for AD patients, using an online detection system.^22^ At set times (5, 10, 15, 20, 40, 50 and 60 min), continuous sampling was interrupted briefly for the collection of manual blood samples (5–7 mL each) to estimate the plasma-to-whole-blood ratios and to measure plasma metabolite fractions. Manual blood samples were collected in heparin tubes and centrifuged for 5 min at 5000 r/min. Plasma was separated from blood cells, and about 1 mL was diluted with 2 mL water and loaded onto a tC2 Sep-Pak cartridge (Waters, Milford, MA), which was pre-activated by elution with 6 mL of methanol and 12 mL of water, respectively. The cartridge was washed with 3 mL water to collect the polar radioactive fraction. Thereafter, the tC18 Sep-Pak cartridge was eluted with 2 mL of methanol and 2 mL of water at Amsterdam UMC and with 1.5 ml of methanol supplemented with 0.1% diisopropylamine, followed by 0.7 ml of water at UMCG to collect the fraction with intact tracer. This fraction was further analysed by HPLC using an Ultimate 3000 system (Dionex, Sunnyvale, CA) equipped with a 1 mL loop at Amsterdam UMC, and at UMCG, a Waters HPLC pump was used for this purpose equipped with a 2 ml loop. As a stationary phase, a Gemini C18, 250 × 10 mm, 5 µm (Phenomenex, Torrance, CA) was used. At Amsterdam UMC, the mobile phase was a gradient of A = acetonitrile and B = 0.1% diisopropylamine in water. The gradient ran for 15 min, decreasing the concentration of eluent B from 80% to 40% in 4 min, followed by 8 min of elution with 40% B at a flow rate of 3 mL min^−1^. At UMCG, the mobile phase consisted of water/acetonitrile/diisopropylamine (55/45/0.1); isocratic elution at 3 ml/min. The eluent was collected in 30 s fractions with a fraction collector, and the fractions were counted for radioactivity using a Wallac 2470 gamma counter (Perkin Elmer, Waltham, MA).

### Data analysis

Structural 3D T1-weighted MRI images were co-registered to the PET images using Vinci v 2.56 software. The Hammers template,^23^ which is incorporated in PVElab,^24^ was used to delineate regions of interest (ROIs) on the co-registered MR scan and superimposed onto the dynamic PET scan to obtain regional time-activity curves (TACs). Online arterial blood TACs were calibrated and corrected for plasma to whole blood ratios, radiolabeled metabolites and delay, using the information from manual blood samples. Eventually, individual metabolite-corrected plasma input functions were generated. Various compartmental models^25^ were used to fit the regional TACs: single tissue reversible (1T2k) and two-tissue irreversible (2T3k) and reversible (2T4k) compartmental models, with and without blood volume (V_B_) as additional fit parameter. To determine the optimal pharmacokinetic model for in vivo kinetics of [^11^C]UCB-J, the Akaike information criterion (AIC)^26^ was used. Furthermore, the SRTM^27^ was assessed by comparing SRTM-derived binding potential (BP_ND_) with plasma input derived distribution volume ratio (DVR). The white-matter centrum semi-ovale (SO) was manually defined using an in-house built tool and was considered as a reference region. Mean SO VOI size was 5.4 ± 2.5 cc for HCs and 4.0 ± 2.6 cc for AD patients. SUVr using three time intervals (40–60, 50–60 for both groups; and 70–90 min only for HCs) were also evaluated. SUVr values obtained from these time intervals were compared with corresponding plasma input derived DVR values.

TRT of micro-parameters (in particular the rate constant from blood to tissue K_1_) and macro-parameters (distribution volume V_T_, DVR and BP_ND_) was evaluated for the preferred model as well as the TRT of R_1_ and BP_ND_ obtained from SRTM. In addition, the effect of scan duration on model preferences, parameter estimation and TRT was assessed. Finally, a separate comparison between HCs and AD patients was performed to assess group differences in regional [^11^C] UCB-J binding.

### Statistical analysis

Statistical analyses were performed using SPSS version 20.0.0 (IBM Corp., Armonk, NY). To assess demographic, clinical and neuroimaging (i.e. SRTM-derived BP_ND_ group comparisons) data, *χ*^2^ tests for discrete variable, t-test and f-tests for continuous data were used. *P* values smaller than 0.05 were considered as significant. TRT was calculated using equation (1), and variability was also assessed by measuring the difference between the test and retest parametric values. Furthermore, the intraclass correlation coefficient (ICC) was analysed using an average-measurement, absolute-agreement, two-way mixed-effects model for each parameter of interest

(1) $$\text{TRT}(\%)=\frac{(\mathrm{Retest}\;\mathrm{value}-\mathrm{Test}\;\mathrm{value})\;}{(\mathrm{Retest}\;\mathrm{value}\;\pm \;\mathrm{Test}\;\mathrm{value})\;}\times 200$$

## Results

The clinical and demographic data are presented in Supplementary Table 1. Net-injected doses were comparable between groups and between test and retest scans (all *p* values > 0.05). There were no significant differences observed in age and gender between AD patients and HCs (*p* > 0.05). As expected, AD patients had a significantly lower MMSE score compared to HCs (*p* < 0.01). Please note that the results from UMCG are presented in the supplementary figures.

### Kinetic analysis

After interim review (i.e. after completion of the HC data), scan duration was reduced to 60 min for subsequent AD subjects. Therefore, all the results, unless specified otherwise, are based on the 60 min PET scan data.

**Figure 1.**
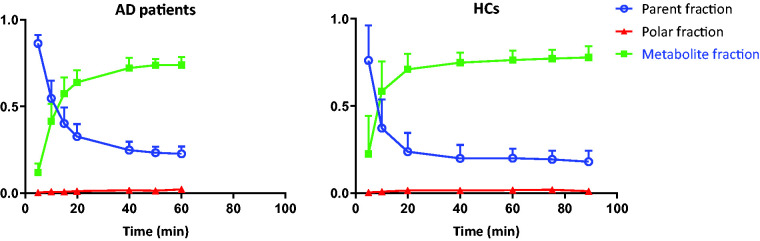
[^11^C]UCB-J metabolite fractions (mean ± SD) in arterial plasma at different time points.

**Figure 2.**
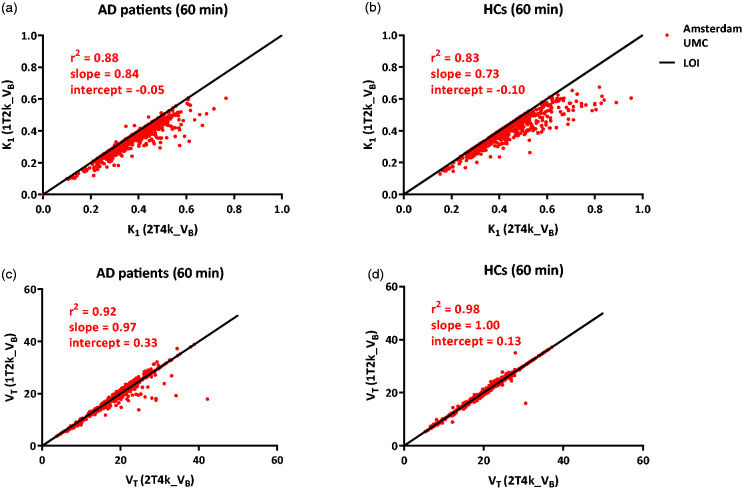
Comparison of K_1_ and V_T_ derived from 1T2k_V_B_ model against K_1_ and V_T_ derived from 2T4k_V_B_ model apart for (a), (c) AD patients and (b), (d) HCs using Amsterdam UMC data. LOI: line of identity.

[^11^C]UCB-J metabolized relatively fast in the plasma with parent fractions of about 60% at 5 min to only 20% after 55 min post-injection (Figure 1). Based on AIC, both 1T2k_V_B_ and 2T4k_V_B_ fitted the [^11^C]UCB-J regional TACs equally well (Supplementary Fig 1). Although in case of the 2T4k_V_B_ model, high standard errors (>25%) were observed for binding potential (BP_ND_ = k_3_/k_4_) estimates. Moreover, both K_1_ (HC: *r*^2^ = 0.83, slope = 0.73; AD: *r*^2^ = 0.88, slope = 0.84) and V_T_ (HC: *r*^2^ = 0.98, slope = 1.00; AD: *r*^2^ = 0.92, slope = 0.97) values correlated well between the two models using 60 min data, suggesting that a 1T2k_V_B_ model is sufficient to assess [^11^C]UCB-J in vivo kinetics (Figure 2). UMCG data also showed a good correspondence between both models (Supplementary Fig 2). K_1 values_ derived from the 1T2k_V_B_ model ranged from 0.16 ± 0.20 mL/cm^3^ in the white-matter SO to 0.39 ± 0.06 mL/cm^3^ in the whole brain grey matter. V_T_ (1T2k_V_B_ model) ranged from 5.36 ± 0.77 for the white-matter SO to 19.32 ± 2.59 for the whole brain grey matter. The V_T_ values for the SO were considerably lower than the examined grey matter regions, and there was no significant difference between AD patients and HCs (*p* = 0.29).

**Figure 3.**
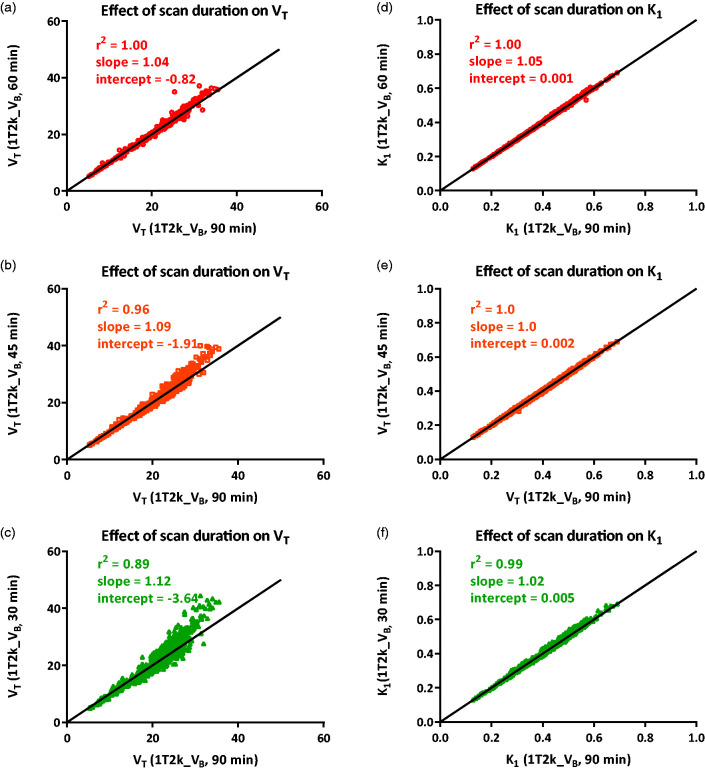
Effect of scan duration reduction on (a), (b), (c) V_T_ and (d), (e), (f) K_1_ estimations using Amsterdam UMC data.

Effect of the scan duration on K_1_ and V_T_ values was estimated using eight HCs obtained at Amsterdam UMC. There was an excellent correlation between K_1_ and V_T_ values obtained from the 60 min scan and the 90 min scan (K_1_: *r*^2^ = 1.00, slope = 1.05; V_T_: *r*^2^ = 1.00, slope = 1.04). Shortening the scan duration to 45 and 30 min showed less reliable results, and an overestimation of the V_T_ values was observed (Figure 3). These results suggest that shortening the scan duration to 60 min has negligible effects on K_1_ and V_T_ values and is sufficient to obtain reliable estimates.

**Figure 4.**
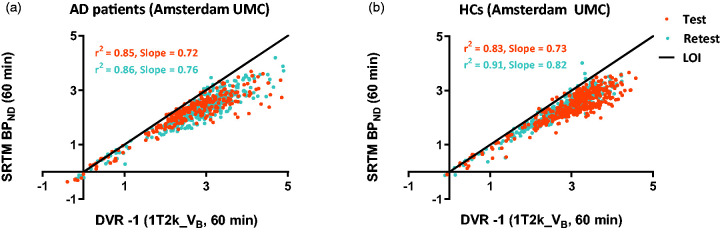
Comparison of SRTM-derived BP_ND_ against plasma input DVR for (a) AD patients and (b) HCs using Amsterdam UMC data. LOI: line of identity.

BP_ND_ values derived from SRTM showed good correlations with plasma input derived DVR values for both AD patients (test: *r*^2^ = 0.85, slope = 0.72; retest: *r*^2^ = 0.86, slope = 0.76) and HCs (test: *r*^2^ = 0.83, slope = 0.73; retest: *r*^2^ = 0.91, slope = 0.82) (see Figure 4). However, an underestimation of approximately 25% was observed. Data from UMCG showed similar results (Supplementary Fig 3).

SUVr plots for test and retest for HC and AD patients are shown in Supplementary Fig 4. The correspondence between SUVr_40–60min_ and SUVr_50–60_ with plasma input derived DVR is presented in Supplementary Fig 5 for AD patients and HCs. SUVr_40–60_ showed a slightly higher correlation with DVR (plasma input) for both AD patients (test: *r*^2^ = 0.83, slope = 0.65; retest: *r*^2^ = 0.89, slope = 0.71) and HCs (test: *r*^2^ = 0.90, slope = 0.73; retest: *r*^2^ = 0.84, slope = 0.74) compared to SUVr_50–60_ (AD: test: *r*^2^ = 0.78, slope = 0.66; retest: *r*^2^ = 0.73, slope = 0.67/HCs: test: *r*^2^ = 0.86, slope = 0.75; retest: *r*^2^ = 0.83, slope = 0.77). SUVr_70–90min_ was also assessed for HCs and had a better correspondence with DVR (plasma input) compared to earlier time intervals (test: *r*^2^ = 0.88, slope = 0.90; retest: *r*^2^ = 0.90, slope =0.90, Supplementary Fig 6). All aforementioned correlations were statistically significant (*p* < 0.05).

### Test–retest analysis

**Figure 5.**
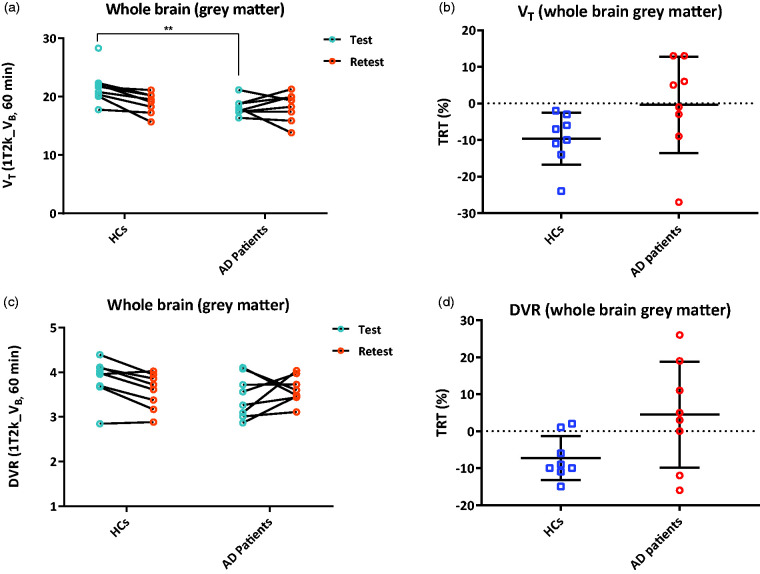
(a), (c) Whole brain (grey matter) V_T_ and plasma input DVR values derived from 1T2k_V_B_ model are displayed for the test and retest scan apart for HCs and AD patients; (b), (d) TRT for whole brain (grey matter) V_T_ and plasma input DVR values are displayed apart for HCs and AD patients using Amsterdam UMC data. Error bars indicate mean ± SD. **implies *p* < 0.01.

**Figure 6.**
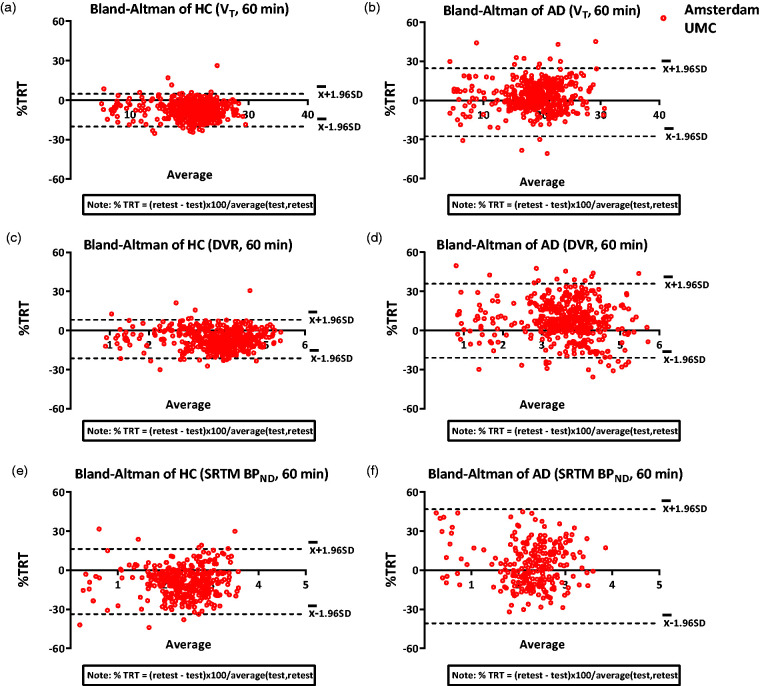
Bland Altman %TRT plots for V_T_, SRTM BP_ND_ and plasma input DVR obtained using 60 min PET data for (a), (c), (e) HCs and (b), (d), (f) AD patients using Amsterdam UMC data.

Whole brain grey matter TRT for V_T_, plasma input DVR and SRTM BP_ND_ were –2.2% ± 8.5, 0.4% ± 12.0 and –8.0% ± 10.2, respectively, averaged over all subjects. For HC subjects, whole brain grey matter TRT for V_T_, plasma input DVR and SRTM BP_ND_ were –7.7% ± 4.3, –6.6% ± 6.1 and –8.2% ± 9.6, respectively (Figure 5). For AD patients, the whole brain grey matter TRT for the kinetic parameters V_T_, plasma input DVR and SRTM BP_ND_ were –3.4% ± 8.1, 7.5% ± 12.7 and –7.6% ± 13.4, respectively. Figure 6 (Supplementary Fig 7 for UMCG data) and Supplementary Fig 8 display the Bland Altman plots for HCs and AD patients, for V_T_, DVR (plasma input) and SRTM BP_ND_ using 60 min data. The Bland Altman plots for 90 min HC data is presented in Supplementary Fig 9. For most of the regions, the TRT was less than 15% (1 SD) for V_T_, plasma input DVR and SRTM BP_ND_. Furthermore, all parameters were systematically lower for most of the Hammers regions in HC in the retest scan compared to the test scan. TRT values for a few ROI are illustrated in Table 1.

**Table 1.** Test and retest values for each kinetic parameter using Amsterdam UMC data.

	HC												AD											
	V_T_			DVR (plasma input)			SRTM BP_ND_			SRTM R_1_			V_T_			DVR (plasma input)			SRTM BP_ND_			SRTM R_1_		
	Test	Retest	%TRT	Test	Retest	%TRT	Test	Retest	%TRT	Test	Retest	%TRT	Test	Retest	%TRT	Test	Retest	%TRT	Test	Retest	%TRT	Test	Retest	%TRT
Hippocampus	20.6 (3.0)	17.8 (1.1)	–1.1 (6.2)	3.6 (0.4)	3.2 (0.4)	–8.8 (6.0)	2.3 (0.4)	1.9 (0.3)	–14.4 (14.4)	1.9 (0.2)	1.9 (0.2)	–3.7 (8.4)	16.3 (1.3)	16.8 (1.8)	3.3 (9.1)	3.0 (0.4)	3.2 (0.3)	7.4 (14.8)	2.0 (0.1)	1.3 (0.6)	–19.5 (-)	1.8 (0.2)	1.9 (0.2)	3.6 (9.1)
Medial Temporal Lobe	21.4 (2.8)	18.8 (1.0)	–8.2 (4.2)	3.7 (0.4)	3.4 (0.4)	–7.2 (5.8)	3.2 (0.3)	3.0 (0.4)	–10.2 (11.1)	1.8 (0.2)	1.7 (0.2)	–2.9 (9.3)	17.7 (1.6)	18.3 (2.1)	3.4 (9.4)	3.3 (0.5)	3.5 (0.3)	7.5 (16.2)	2.4 (1.3)	3.1 (0.5)	–0.3 (21.8)	1.7 (0.2)	1.8 (0.2)	4.1 (9.4)
Cerebellum	18.1 (1.8)	16.4 (1.3)	–7.2 (4.9)	3.2 (0.3)	3.0 (0.4)	–6.1 (7.7)	1.8 (0.5)	1.7 (0.5)	–10.9 (12.1)	2.5 (0.1)	2.1 (0.9)	–2.8 (6.7)	15.5 (1.4)	16.3 (1.9)	4.7 (9.0)	2.9 (0.3)	3.1 (0.3)	8.8 (12.8)	1.3 (0.7)	1.7 (0.5)	15.8 (16.6)	1.7 (1.2)	2.4 (0.6)	7.6 (14.8)
Brainstem	8.4 (0.9)	7.9 (1.0)	–4.8 (4.2)	1.5 (0.2)	1.4 (0.2)	–3.7 (5.4)	0.5 (0.2)	0.4 (0.2)	–11.8 (17.8)	1.4 (0.6)	1.6 (0.2)	–2.7 (6.6)	7.4 (0.7)	7.5 (0.8)	1.4 (12.3)	1.4 (0.1)	1.4 (0.1)	5.5 (7.7)	0.4 (0.1)	0.5 (0.1)	10.7 (18.2)	1.7 (0.1)	1.5 (0.7)	0.9 (8.4)
Caudate Nucleus	22.9 (3.3)	20.6 (2.3)	–7.6 (6.8)	3.9 (0.5)	3.7 (0.5)	–7.6 (6.8)	2.5 (0.4)	2.2 (0.4)	–9.2 (5.9)	2.3 (0.3)	2.2 (0.5)	–0.8 (6.5)	19.2 (2.2)	20.5 (3.0)	10.3 (17.8)	3.6 (0.5)	3.9 (0.6)	10.3 (17.8)	2.1 (0.5)	2.2 (0.4)	9.9 (22.4)	2.1 (0.3)	2.1 (0.2)	2.0 (10.9)
Putamen	28.7 (3.6)	25.8 (1.2)	–5.5 (4.4)	4.9 (0.4)	4.7 (0.5)	–5.5 (4.4)	3.3 (0.3)	3.0 (0.4)	–5.3 (8.0)	2.8 (0.2)	2.7 (0.3)	–2.0 (5.7)	25.9 (3.2)	26.9 (2.9)	–3.9 (8.2)	4.8 (0.5)	5.2 (0.5)	3.9 (17.8)	3.1 (0.3)	3.3 (0.4)	3.8 (16.1)	2.8 (0.3)	2.9 (0.4)	3.2 (9.5)
Thalamus	19.8 (3.5)	17.7 (2.7)	–5.8 (5.4)	3.5 (0.6)	3.2 (0.6)	–6.6 (5.4)	2.2 (0.6)	2.0 (0.5)	–7.3 (11.6)	2.6 (0.3)	2.5 (0.3)	–5.4 (7.1)	14.8 (0.9)	16.0 (2.3)	7.2 (16.3)	2.8 (0.5)	3.1 (0.5)	7.2 (16.3)	1.6 (0.5)	1.6 (0.6)	9.4 (12.4)	2.0 (0.9)	2.2 (0.4)	–0.5 (13.1)
Whole Brain (grey matter)	21.9 (3.0)	19.4 (1.3)	–7.7 (4.3)	3.8 (0.4)	3.5 (0.4)	–6.6 (6.1)	2.4 (0.3)	2.2 (0.4)	–8.2 (9.6)	2.4 (0.2)	2.3 (0.3)	–1.9 (6.3)	18.2 (1.5)	18.9 (1.8)	3.4 (8.1)	3.4 (0.4)	3.6 (0.3)	7.5 (12.7)	2.3 (0.1)	2.2 (0.3)	–7.6 (13.4)	2.3 (0.2)	2.3 (0.2)	1.6 (10.0)

Note: values indicate mean and SD in brackets.

Figure 5 shows that there was a significant difference between HCs and AD patients in whole brain grey matter when using test scans and V_T_ as a parameter of interest (*p* = 0.007). There was one HC subject that appeared as an outlier (>2 SD) in Figure 5(a), the significant difference remained (*p* = 0.003) even if this subject was excluded from the analysis. However, the significant difference was lost when the V_T_ values estimated from the retest scans were used for this assessment (*p* = 0.54). Furthermore, lower plasma-input DVR values (trend, 0.05 > *p* > 0.1) were observed in AD patients when compared to HC subjects for test scans (*p* = 0.09, Figure 5(c)). Here again, there was one HC subject with low plasma input DVR values appearing to be an outlier (<2 SD), when this subject was excluded from analysis, a significant difference was observed between AD patients and HCs (*p* = 0.01). This was not the case when the retest scans were used for the same analysis (*p* = 0.41). In addition, no significant difference between the groups was observed in neither the test nor the retest scans when using SRTM BP_ND_ as the parameters of interest (*p* = 0.41; *p* = 0.76). Furthermore, a significant difference was observed between the test and retest scan for whole brain grey matter V_T_ in HCs (*p* = 0.03). This was not observed for AD patients.

The comparisons of V_T_, plasma input DVR and SRTM BP_ND_ between HCs and AD patients for hippocampus, medial temporal lobe and whole brain grey matter are presented in Supplementary Fig 10. Significant reductions of 20.9%, 17.3% and 16.9% in V_T_ were observed for AD patients for the regions: hippocampus (AD: 16.3 ± 1.3; HC: 20.6 ± 3.0), MTL (AD: 17.7 ± 1.6; HC: 21.4 ± 2.8) and whole brain grey matter (AD: 18.2 ± 1.5; HC: 21.9 ± 3.0) when the test scan was used for the analysis (all *p* < 0.01), respectively. When the retest scan was used for the same analysis, no significant reduction was observed for these regions (all *p* > 0.05). However, using the average V_T_ of the test and retest scan, a significant reduction of 11.8% in V_T_ was observed in the hippocampus for AD patients (AD: 16.5 ± 1.4; HC: 18.7 ± 1.2) (*p* = 0.008). A trend was observed in case of MTL and whole-brain V_T_, hippocampus DVR and hippocampus BP_ND_ (Supplementary Fig 10). The ICC for V_T_, DVR (plasma input) and SRTM BP_ND_ was 0.94, 0.92 and 0.92 with 95% confident interval of 0.93–0.95, 0.90–0.93 and 0.91–0.94, respectively.

Whole brain grey matter TRT averaged across all subjects for SUVr_40–60min_ and SUVr_50–60min_ were 1 ± 8.2% and 2 ± 8.2%, respectively. For HCs, whole brain grey matter TRT for SUVr_40–60min_ was –3% ± 5.0 and 5% ± 9.1 for AD patients. Furthermore, whole brain grey matter TRT for SUVr_50–60min_ was 0% ± 5.4 for HCs and 4% ± 10.3 for AD patients. The Bland Altman plots for the SUVr_40–60min_ and SUVr_50–60min_ for each subject group are presented in Supplementary Fig 11. Supplementary Fig 12 illustrates the whole brain (grey matter) SUVr values for test and retest for HCs and AD patients. No significant difference between AD patients and HCs in neither the test nor the retest scan in whole brain grey matter when using SUVR_40–60min_ (*p* = 0.13 for test, *p* = 0.97 for retest) or SUVR_50–60min_ (*p* = 0.45 for test, *p* = 0.85 for retest) was observed.

## Discussion

The current study investigated the kinetic analysis and the TRT of the regional pharmacokinetic parameters of [^11^C]UCB-J, a PET tracer binding to SV2A in the brain. An one-tissue compartment model with a blood volume parameter (1T2k_V_B_) was sufficient to describe the in vivo kinetics of [^11^C]UCB-J. In addition, SRTM could also be used to quantify [^11^C]UCB-J in a non-invasive manner. Furthermore, we observed a mean 28-day TRT for, V_T_, plasma input derived DVR and SRTM BP_ND_ less than 15% (1SD) averaged over all subjects, implying that, in case of intervention studies or drug trials, [^11^C]UCB-J can be used to quantify the impact of the drug on SV2A, if the effect size of the drug is higher than 15%.

The present findings with regard to the model preference are in line with another recent [^11^C]UCB-J study.^15^ It was observed that both 1T2k_V_B_ and 2T4k_V_B_ compartment models had competing model preferences based on AIC. However, the k_3_/k_4_ (BP_ND_) estimates from the 2T4k_V_B_ model were highly unreliable. Moreover, K_1_ and V_T_ estimates between the 1T2k_V_B_ and 2T4k_V_B_ models were highly correlated as is illustrated in Figure 2 and Supplementary Fig 2. This suggested that the use of 1T2k_V_B_ model is sufficient to evaluate the in vivo kinetics of [^11^C]UCB-J. Furthermore, a decrease in the scan duration from 90 to 60 min also had no significant effect neither on the model preferences nor on the parameter estimations.

No significant difference was observed in the SO V_Ts_ between the HCs and AD patients, supporting the use of this region as a reference region. However, the kinetic behaviour of the tracer in the region is quite different from the rest of the brain regions. The influx of the tracer in SO was much lower than in other brain regions, resulting in a higher R_1_ value than usual (Table 1). One of the assumptions for the implementation of the SRTM is that the non-specific compartment in the reference region and the target region should be equal, which is probably not fulfilled when using SO as reference region. A recent study illustrated a higher non-displaceable distribution volume (V_ND_) estimation when using SO as reference region, suggesting an underestimation of the specific signal when performing SRTM analysis.^28^ This indicates that SO might not be an ideal reference region but could be used as a normalization region. Further studies are necessary to validate the use of SO or other possible reference regions for proper quantification of [^11^C]UCB-J.

A good correlation between SRTM BP_ND_ values and plasma input DVR values was observed, but with approximately 25% underestimation. A possible explanation for this phenomenon could be that V_B_ correction is not present in the SRTM model since it is assumed that the V_B_ is rather constant between the different brain regions. This might not be true for all tracers and all regions, particularly in the present situation, where the reference region seems to have different kinetics (lower tracer influx; K_1_) than the grey matter ROIs. Another reason could be the significantly lower activity in the SO, which resulted in noisy reference region TACs, which in turn led to fitting errors (high standard errors for estimated parameters) in case of SRTM. V_T_ values are in general more forgiving in this aspect and probably; therefore, plasma input DVRs were slightly immune to the noise in the reference TACs. The observed TRT values were high for reference region-based methods, and this could partially be explained by variability in the SO definition.

Although a clear trend of lower regional parametric values in the AD patients was observed when compared to HCs, a significant difference was observed in the hippocampus, MTL and whole brain grey matter only when using test scans and, the average of test and retest scans V_T_s as the parameter of interest. The significance remained even after excluding a possible outlier (by visual interpretation of the plots) from this analysis. This HC subject had a very high V_T_ value for the corresponding regions, but nothing specifically different/erroneous was observed in the data. Possibly, the subject has a high physiological uptake; unfortunately, there was no retest data available for this subject to assess this aspect. Similar comparisons using the retest scans presented no significant differences between groups (Supplementary Fig 10). SRTM BP_ND_ values for HCs were higher in both, the test and the retest scan when compared to AD patients. However, this difference was less pronounced in the retest scan. Unfortunately, it was not possible to use all the values from each subject for this parameter, since there was a high uncertainty observed when estimating SRTM BP_ND_ (as discussed earlier). This could also explain the higher inter-subject variability in both groups when using SRTM BP_ND_ compared to other parameters.

The current study observed a significant reduction of 20.9% in hippocampus V_T_ for AD patients when compared to HCs. An earlier study by Chen et al.^17^ observed a higher reduction of SV2A in the hippocampus, namely a reduction of 28% in hippocampus V_T_. The AD patients in the current study were on average 10 years younger than the AD group in the study by Chen et al.^17^ This could explain the lower percentage reduction in the hippocampus in the current study. Furthermore, the significant reduction of SV2A in the hippocampus was only observed in the test scan and in the average of test and retest scan. Moreover, we also observed a trend (0.05 > *p* > 0.1) in hippocampus when using SRTM BP_ND_ values from the retest and when using the average of SRTM BP_ND_ values from test and retest scans. Chen et al.^17^ also observed a significant reduction of synaptic density in AD patients when SRTM2 BP_ND_ was used as the parameter of interest.

The observed difference in results based on the use of test or retest data could be the result of the systematically lower parametric values for the retest scans when compared to test scans in this study. Other TRT studies of [^11^C]UCB-J imaging did not show a systematic bias in one of the parameters.^15^ In contrast to our study, previous studies, that reported no significant parameter estimation bias, performed the test and retest scans on the same day. The reason for finding a negative bias in the retest scan is still unclear. No technical errors (e.g. related to data acquisition or data processing), diurnal variations or changes in food intake were detected that could explain this underestimation. The AUCs of the SUV whole brain and metabolite corrected SUV plasma were also compared between test and retest for both HCs and AD patients. No significant difference in the SUV whole brain uptake (*p* = 0.13) nor the metabolite corrected SUV plasma activity (*p* = 0.80) was observed for HCs between test and retest scans. Similarly, AD patients also had no significant difference in SUV brain uptake (*p* = 0.23) or metabolite-corrected SUV plasma activity (*p* = 0.92). This also illustrates that the cause of systemic bias is not technical but most likely biological. A possible hypothesis is that there is an increase in the number of synaptic vesicles that are already transported to the membrane and ready to be released, also called the readily releasable pool (RRP) of vesicles.^29^ The increase in the number of synaptic vesicles could be true for the test scan due to stress of undergoing the procedure for the first time. The stress levels would be lower for the second scan because the subjects were already familiar with the procedure, in particular for HC who are more aware of the situation. However, we do not know the level of acute stress for the first PET scan.

It should be noted that bias between test and retest scans is not uncommon. The current study is not the first study that observed a negative bias in the retest scan. This was also observed in another study by Kim et al..^30^ They found that the retest BP_ND_ values were 6% lower across all regions for [^11^C]DASB, which is a tracer targeting serotonin transporters. The researchers gave several possible explanations for this observation. One of the explanations was that the negative bias in the retest could be attributed to acute stress. Acute stress activates several physiologic systems, which leads to higher cortisol levels which modulate the serotonergic neuronal activity.^31,32^ Furthermore, Leurquin-Sterk et al.^33^ also observed a negative bias in the retest scan for V_T_ values for ^18^F‐FPEB, which binds to metabotropic glutamate subtype 5 receptor (mGluR5). Their explanation was that V_T_ does not separate free and nonspecific compartments from the specific compartment and, therefore, more sensitive to errors in the input function. The researchers did not observe the negative bias for BP_ND_ (k_3_/k_4_). In the current study, the negative bias was observed in all the parameter estimations but only for HCs (V_T_, plasma-input derived DVR and SRTM BP_ND_).

In case of the SUVr values, a high TRT was observed irrespective of the time points (50–60 min or 40–60 min). However, no clear difference between the patient and control group was observed suggesting that this parameter might still be dominated by the signal from the nonspecific compartment. It could also be the case that SUVr did not reach equilibrium using earlier time-points, as we saw a negative bias of ±25% for HCs and even more for AD patients. The negative bias decreased to 10% using SUVr_70–90min_ for HCs. Since there was only 60 min data available for AD patients, it was not possible to perform group comparisons for SUVr_70–90min_. Further studies should focus on assessing later time points in AD patients to assess the applicability of SUVr_70–90min_.

An initial aim of the study was to validate the [^11^C]UCB-J kinetics irrespective of differences between the centres, i.e. to explore the generalizability of the findings in a multicentre setting. Different scanners were used in both centres, and there were subtle differences in the metabolite measurement methodology between the centres which can cause variability in the results. Certain variability was observed in results of UMCG and Amsterdam UMC data. As can be seen from Figure 6 and Supplementary Fig 7, the main difference was observed in case of V_T_ values. A small non-significant difference was observed in the whole blood activity over time in case of UMCG blood data when compared to the Amsterdam UMC blood data, which could lead to some differences in parametric estimations. However, it is difficult to conclude that if these differences can cause significant variability in the parameter estimations due to the small sample size of UMCG data. However, it is important to note that the results and conclusions do not differ with or without the inclusion of UMCG data.

## Conclusion

[^11^C]UCB-J kinetics can be well described by a reversible single tissue compartment model with V_B_ fraction as a fit parameter. Reliable fits can be obtained with a 60 min scan duration for both plasma input and reference tissue models, which is in line with an earlier study. The current study observed a mean 28-day TRT for V_T_, plasma input derived DVR and SRTM-derived BP_ND_ of <15% (1 SD) averaged over all subjects, indicating adequate repeatability of [^11^C]UCB-J, which is important for longitudinal studies and clinical trials. SRTM-derived BP_ND_ correlates well with plasma input derived DVR, although some negative bias in both HC and AD subjects is seen.

## Supplemental Material

sj-pdf-1-jcb-10.1177_0271678X20964248 - Supplemental material for Kinetics and 28-day test–retest repeatability and reproducibility of [^11^C]UCB-J PET brain imaging Click here for additional data file. Supplemental material, sj-pdf-1-jcb-10.1177_0271678X20964248 for Kinetics and 28-day test–retest repeatability and reproducibility of [^11^C]UCB-J PET brain imaging by Hayel Tuncel, Ronald Boellaard, Emma M Coomans, Erik FJ de Vries, Andor WJM Glaudemans, Paula Kopschina Feltes, David V García, Sander CJ Verfaillie, Emma E Wolters, Steven P Sweeney, J Michael Ryan, Magnus Ivarsson, Berkley A Lynch, Patrick Schober, Philip Scheltens, Robert C Schuit, Albert D Windhorst, Peter P De Deyn, Bart NM van Berckel and Sandeep SV Golla in Journal of Cerebral Blood Flow & Metabolism

sj-pdf-2-jcb-10.1177_0271678X20964248 - Supplemental material for Kinetics and 28-day test–retest repeatability and reproducibility of [^11^C]UCB-J PET brain imaging Click here for additional data file. Supplemental material, sj-pdf-2-jcb-10.1177_0271678X20964248 for Kinetics and 28-day test–retest repeatability and reproducibility of [^11^C]UCB-J PET brain imaging by Hayel Tuncel, Ronald Boellaard, Emma M Coomans, Erik FJ de Vries, Andor WJM Glaudemans, Paula Kopschina Feltes, David V García, Sander CJ Verfaillie, Emma E Wolters, Steven P Sweeney, J Michael Ryan, Magnus Ivarsson, Berkley A Lynch, Patrick Schober, Philip Scheltens, Robert C Schuit, Albert D Windhorst, Peter P De Deyn, Bart NM van Berckel and Sandeep SV Golla in Journal of Cerebral Blood Flow & Metabolism

sj-pdf-3-jcb-10.1177_0271678X20964248 - Supplemental material for Kinetics and 28-day test–retest repeatability and reproducibility of [^11^C]UCB-J PET brain imaging Click here for additional data file. Supplemental material, sj-pdf-3-jcb-10.1177_0271678X20964248 for Kinetics and 28-day test–retest repeatability and reproducibility of [^11^C]UCB-J PET brain imaging by Hayel Tuncel, Ronald Boellaard, Emma M Coomans, Erik FJ de Vries, Andor WJM Glaudemans, Paula Kopschina Feltes, David V García, Sander CJ Verfaillie, Emma E Wolters, Steven P Sweeney, J Michael Ryan, Magnus Ivarsson, Berkley A Lynch, Patrick Schober, Philip Scheltens, Robert C Schuit, Albert D Windhorst, Peter P De Deyn, Bart NM van Berckel and Sandeep SV Golla in Journal of Cerebral Blood Flow & Metabolism

sj-pdf-4-jcb-10.1177_0271678X20964248 - Supplemental material for Kinetics and 28-day test–retest repeatability and reproducibility of [^11^C]UCB-J PET brain imaging Click here for additional data file. Supplemental material, sj-pdf-4-jcb-10.1177_0271678X20964248 for Kinetics and 28-day test–retest repeatability and reproducibility of [^11^C]UCB-J PET brain imaging by Hayel Tuncel, Ronald Boellaard, Emma M Coomans, Erik FJ de Vries, Andor WJM Glaudemans, Paula Kopschina Feltes, David V García, Sander CJ Verfaillie, Emma E Wolters, Steven P Sweeney, J Michael Ryan, Magnus Ivarsson, Berkley A Lynch, Patrick Schober, Philip Scheltens, Robert C Schuit, Albert D Windhorst, Peter P De Deyn, Bart NM van Berckel and Sandeep SV Golla in Journal of Cerebral Blood Flow & Metabolism

sj-pdf-5-jcb-10.1177_0271678X20964248 - Supplemental material for Kinetics and 28-day test–retest repeatability and reproducibility of [^11^C]UCB-J PET brain imaging Click here for additional data file. Supplemental material, sj-pdf-5-jcb-10.1177_0271678X20964248 for Kinetics and 28-day test–retest repeatability and reproducibility of [^11^C]UCB-J PET brain imaging by Hayel Tuncel, Ronald Boellaard, Emma M Coomans, Erik FJ de Vries, Andor WJM Glaudemans, Paula Kopschina Feltes, David V García, Sander CJ Verfaillie, Emma E Wolters, Steven P Sweeney, J Michael Ryan, Magnus Ivarsson, Berkley A Lynch, Patrick Schober, Philip Scheltens, Robert C Schuit, Albert D Windhorst, Peter P De Deyn, Bart NM van Berckel and Sandeep SV Golla in Journal of Cerebral Blood Flow & Metabolism

sj-pdf-6-jcb-10.1177_0271678X20964248 - Supplemental material for Kinetics and 28-day test–retest repeatability and reproducibility of [^11^C]UCB-J PET brain imaging Click here for additional data file. Supplemental material, sj-pdf-6-jcb-10.1177_0271678X20964248 for Kinetics and 28-day test–retest repeatability and reproducibility of [^11^C]UCB-J PET brain imaging by Hayel Tuncel, Ronald Boellaard, Emma M Coomans, Erik FJ de Vries, Andor WJM Glaudemans, Paula Kopschina Feltes, David V García, Sander CJ Verfaillie, Emma E Wolters, Steven P Sweeney, J Michael Ryan, Magnus Ivarsson, Berkley A Lynch, Patrick Schober, Philip Scheltens, Robert C Schuit, Albert D Windhorst, Peter P De Deyn, Bart NM van Berckel and Sandeep SV Golla in Journal of Cerebral Blood Flow & Metabolism

sj-pdf-7-jcb-10.1177_0271678X20964248 - Supplemental material for Kinetics and 28-day test–retest repeatability and reproducibility of [^11^C]UCB-J PET brain imaging Click here for additional data file. Supplemental material, sj-pdf-7-jcb-10.1177_0271678X20964248 for Kinetics and 28-day test–retest repeatability and reproducibility of [^11^C]UCB-J PET brain imaging by Hayel Tuncel, Ronald Boellaard, Emma M Coomans, Erik FJ de Vries, Andor WJM Glaudemans, Paula Kopschina Feltes, David V García, Sander CJ Verfaillie, Emma E Wolters, Steven P Sweeney, J Michael Ryan, Magnus Ivarsson, Berkley A Lynch, Patrick Schober, Philip Scheltens, Robert C Schuit, Albert D Windhorst, Peter P De Deyn, Bart NM van Berckel and Sandeep SV Golla in Journal of Cerebral Blood Flow & Metabolism

sj-pdf-8-jcb-10.1177_0271678X20964248 - Supplemental material for Kinetics and 28-day test–retest repeatability and reproducibility of [^11^C]UCB-J PET brain imaging Click here for additional data file. Supplemental material, sj-pdf-8-jcb-10.1177_0271678X20964248 for Kinetics and 28-day test–retest repeatability and reproducibility of [^11^C]UCB-J PET brain imaging by Hayel Tuncel, Ronald Boellaard, Emma M Coomans, Erik FJ de Vries, Andor WJM Glaudemans, Paula Kopschina Feltes, David V García, Sander CJ Verfaillie, Emma E Wolters, Steven P Sweeney, J Michael Ryan, Magnus Ivarsson, Berkley A Lynch, Patrick Schober, Philip Scheltens, Robert C Schuit, Albert D Windhorst, Peter P De Deyn, Bart NM van Berckel and Sandeep SV Golla in Journal of Cerebral Blood Flow & Metabolism

sj-pdf-9-jcb-10.1177_0271678X20964248 - Supplemental material for Kinetics and 28-day test–retest repeatability and reproducibility of [^11^C]UCB-J PET brain imaging Click here for additional data file. Supplemental material, sj-pdf-9-jcb-10.1177_0271678X20964248 for Kinetics and 28-day test–retest repeatability and reproducibility of [^11^C]UCB-J PET brain imaging by Hayel Tuncel, Ronald Boellaard, Emma M Coomans, Erik FJ de Vries, Andor WJM Glaudemans, Paula Kopschina Feltes, David V García, Sander CJ Verfaillie, Emma E Wolters, Steven P Sweeney, J Michael Ryan, Magnus Ivarsson, Berkley A Lynch, Patrick Schober, Philip Scheltens, Robert C Schuit, Albert D Windhorst, Peter P De Deyn, Bart NM van Berckel and Sandeep SV Golla in Journal of Cerebral Blood Flow & Metabolism

sj-pdf-10-jcb-10.1177_0271678X20964248 - Supplemental material for Kinetics and 28-day test–retest repeatability and reproducibility of [^11^C]UCB-J PET brain imaging Click here for additional data file. Supplemental material, sj-pdf-10-jcb-10.1177_0271678X20964248 for Kinetics and 28-day test–retest repeatability and reproducibility of [^11^C]UCB-J PET brain imaging by Hayel Tuncel, Ronald Boellaard, Emma M Coomans, Erik FJ de Vries, Andor WJM Glaudemans, Paula Kopschina Feltes, David V García, Sander CJ Verfaillie, Emma E Wolters, Steven P Sweeney, J Michael Ryan, Magnus Ivarsson, Berkley A Lynch, Patrick Schober, Philip Scheltens, Robert C Schuit, Albert D Windhorst, Peter P De Deyn, Bart NM van Berckel and Sandeep SV Golla in Journal of Cerebral Blood Flow & Metabolism

sj-pdf-11-jcb-10.1177_0271678X20964248 - Supplemental material for Kinetics and 28-day test–retest repeatability and reproducibility of [^11^C]UCB-J PET brain imaging Click here for additional data file. Supplemental material, sj-pdf-11-jcb-10.1177_0271678X20964248 for Kinetics and 28-day test–retest repeatability and reproducibility of [^11^C]UCB-J PET brain imaging by Hayel Tuncel, Ronald Boellaard, Emma M Coomans, Erik FJ de Vries, Andor WJM Glaudemans, Paula Kopschina Feltes, David V García, Sander CJ Verfaillie, Emma E Wolters, Steven P Sweeney, J Michael Ryan, Magnus Ivarsson, Berkley A Lynch, Patrick Schober, Philip Scheltens, Robert C Schuit, Albert D Windhorst, Peter P De Deyn, Bart NM van Berckel and Sandeep SV Golla in Journal of Cerebral Blood Flow & Metabolism

sj-pdf-12-jcb-10.1177_0271678X20964248 - Supplemental material for Kinetics and 28-day test–retest repeatability and reproducibility of [^11^C]UCB-J PET brain imaging Click here for additional data file. Supplemental material, sj-pdf-12-jcb-10.1177_0271678X20964248 for Kinetics and 28-day test–retest repeatability and reproducibility of [^11^C]UCB-J PET brain imaging by Hayel Tuncel, Ronald Boellaard, Emma M Coomans, Erik FJ de Vries, Andor WJM Glaudemans, Paula Kopschina Feltes, David V García, Sander CJ Verfaillie, Emma E Wolters, Steven P Sweeney, J Michael Ryan, Magnus Ivarsson, Berkley A Lynch, Patrick Schober, Philip Scheltens, Robert C Schuit, Albert D Windhorst, Peter P De Deyn, Bart NM van Berckel and Sandeep SV Golla in Journal of Cerebral Blood Flow & Metabolism

sj-pdf-13-jcb-10.1177_0271678X20964248 - Supplemental material for Kinetics and 28-day test–retest repeatability and reproducibility of [^11^C]UCB-J PET brain imaging Click here for additional data file. Supplemental material, sj-pdf-13-jcb-10.1177_0271678X20964248 for Kinetics and 28-day test–retest repeatability and reproducibility of [^11^C]UCB-J PET brain imaging by Hayel Tuncel, Ronald Boellaard, Emma M Coomans, Erik FJ de Vries, Andor WJM Glaudemans, Paula Kopschina Feltes, David V García, Sander CJ Verfaillie, Emma E Wolters, Steven P Sweeney, J Michael Ryan, Magnus Ivarsson, Berkley A Lynch, Patrick Schober, Philip Scheltens, Robert C Schuit, Albert D Windhorst, Peter P De Deyn, Bart NM van Berckel and Sandeep SV Golla in Journal of Cerebral Blood Flow & Metabolism
