# Supplementary figures and images for: Kinetics and 28-day test–retest repeatability and reproducibility of [11C]UCB-J PET brain imaging
Source: J Cereb Blood Flow Metab. 2020 Oct 8;41(6):1338–50. doi: 10.1177/0271678X20964248 (PMC8138337; doi:10.1177/0271678X20964248)

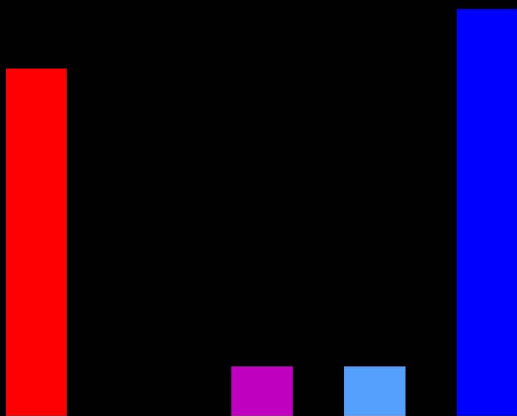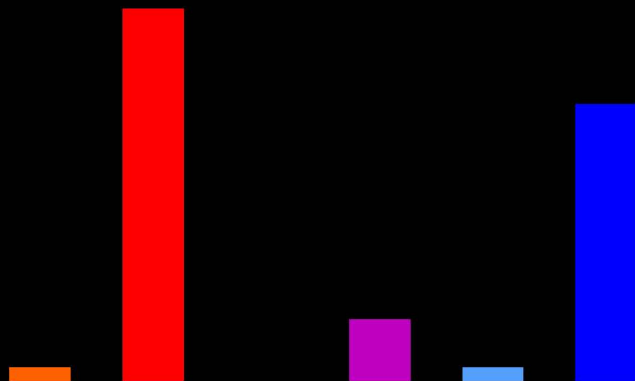

Supplement: sj-pdf-1-jcb-10.1177_0271678X20964248 - Supplemental material for Kinetics and 28-day test–retest repeatability and reproducibility of [11C]UCB-J PET brain imaging [file sj-pdf-1-jcb-10.1177_0271678X20964248.pdf]

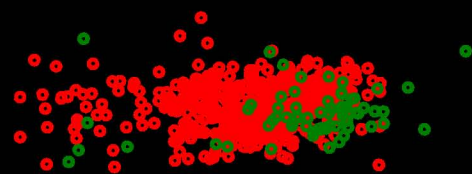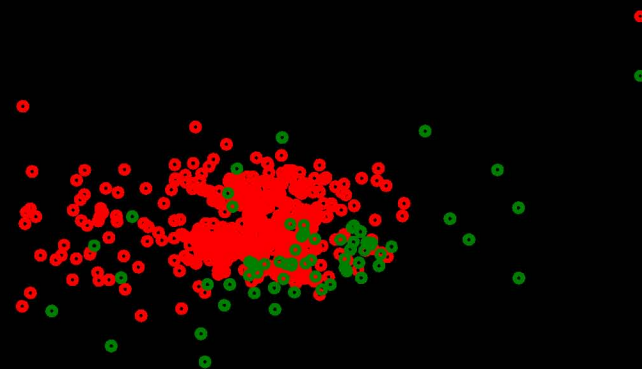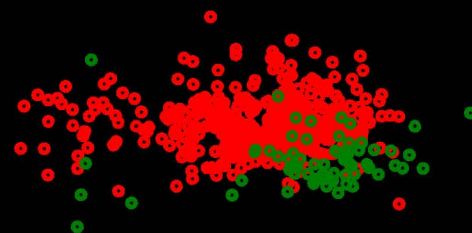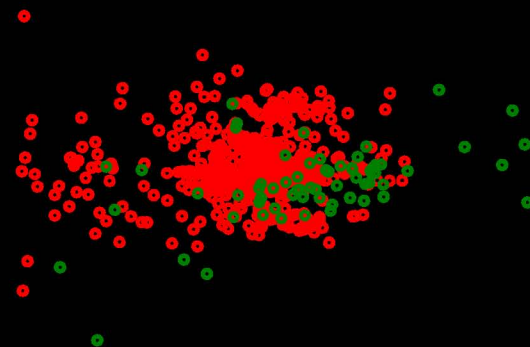

Supplement: sj-pdf-3-jcb-10.1177_0271678X20964248 - Supplemental material for Kinetics and 28-day test–retest repeatability and reproducibility of [11C]UCB-J PET brain imaging [file sj-pdf-3-jcb-10.1177_0271678X20964248.pdf]

$r^2 = 0.93$   
slope = 0.98  
intercept = -0.01

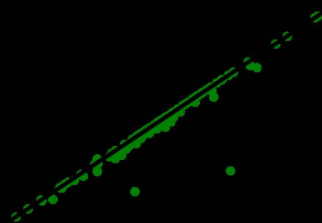

$r^2 = 0.97$   
slope = 0.95  
intercept = 0.84

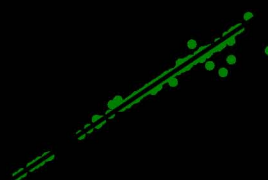

$r^2 = 0.91$   
slope = 0.91  
intercept = 0.03

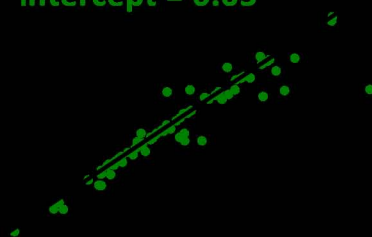

$r^2 = 0.97$   
slope = 0.93  
intercept = 0.01

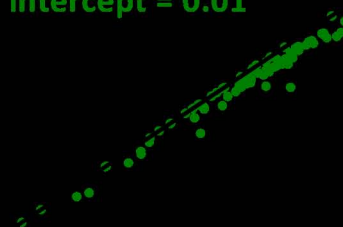

Supplement: sj-pdf-5-jcb-10.1177_0271678X20964248 - Supplemental material for Kinetics and 28-day test–retest repeatability and reproducibility of [11C]UCB-J PET brain imaging [file sj-pdf-5-jcb-10.1177_0271678X20964248.pdf]

$r^2 = 0.93$ , Slope = 0.73

$r^2 = 0.92$ , Slope = 0.66

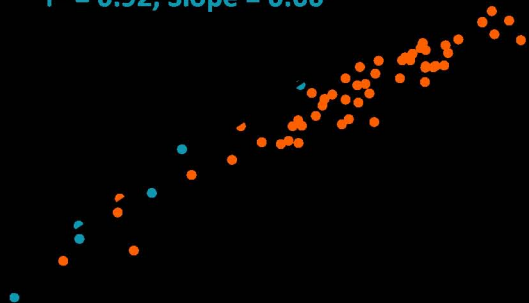

$r^2 = 0.85$ , Slope = 0.84

$r^2 = 0.96$ , Slope = 0.75

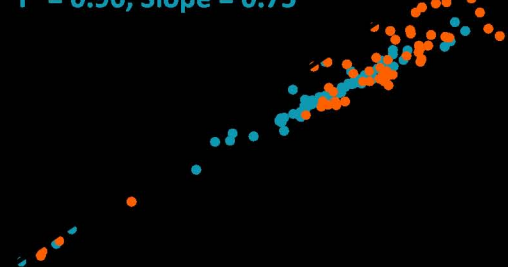

Supplement: sj-pdf-6-jcb-10.1177_0271678X20964248 - Supplemental material for Kinetics and 28-day test–retest repeatability and reproducibility of [11C]UCB-J PET brain imaging [file sj-pdf-6-jcb-10.1177_0271678X20964248.pdf]

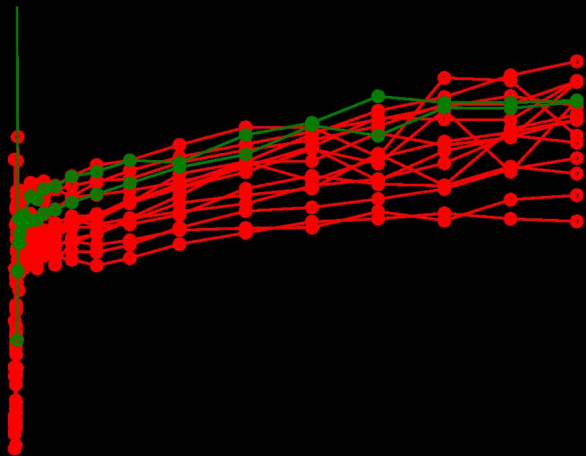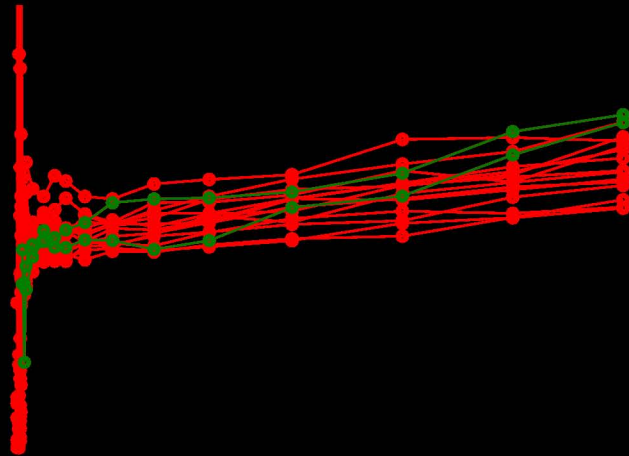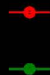

Supplement: sj-pdf-7-jcb-10.1177_0271678X20964248 - Supplemental material for Kinetics and 28-day test–retest repeatability and reproducibility of [11C]UCB-J PET brain imaging [file sj-pdf-7-jcb-10.1177_0271678X20964248.pdf]

$r^2 = 0.88$ , Slope = 0.90

$r^2 = 0.90$ , Slope = 0.90

$r^2 = 0.97$ , Slope = 0.84

$r^2 = 0.95$ , Slope = 0.91

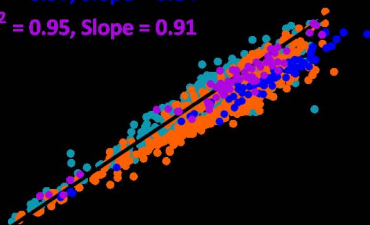

Supplement: sj-pdf-9-jcb-10.1177_0271678X20964248 - Supplemental material for Kinetics and 28-day test–retest repeatability and reproducibility of [11C]UCB-J PET brain imaging [file sj-pdf-9-jcb-10.1177_0271678X20964248.pdf]

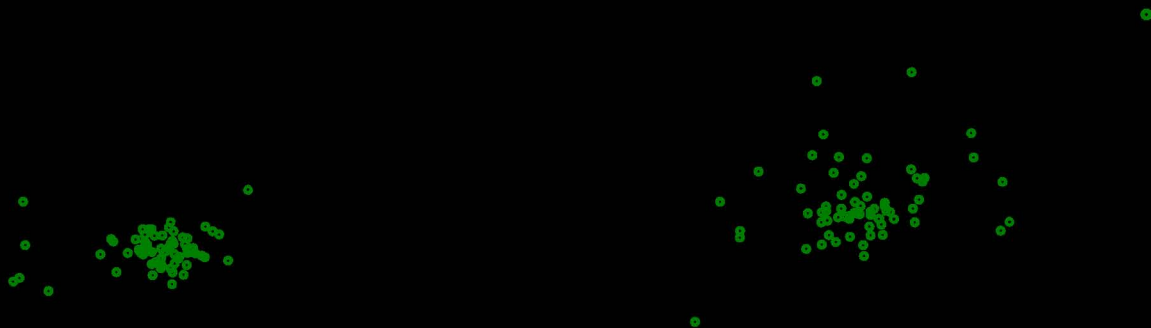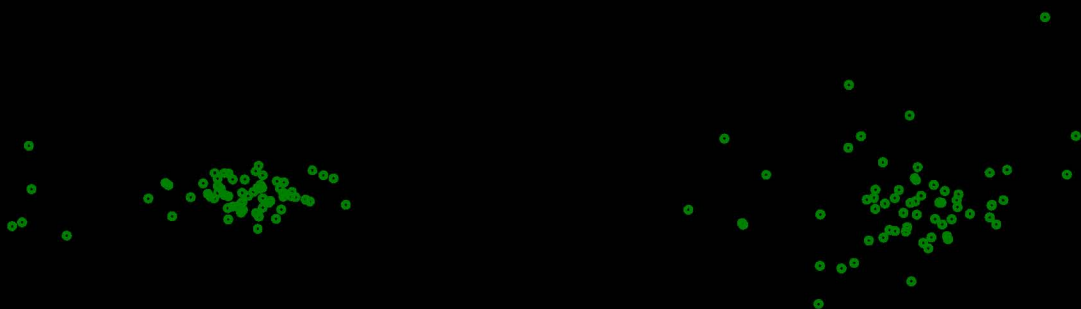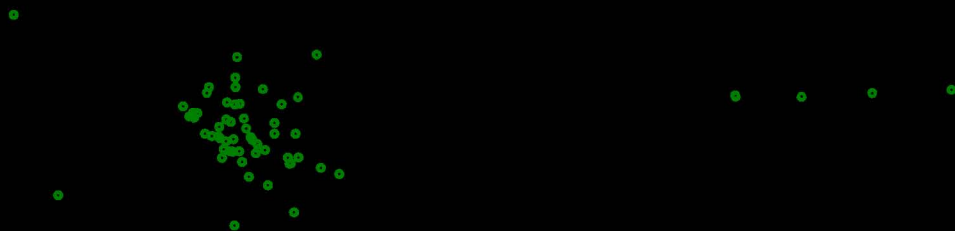

Supplement: sj-pdf-10-jcb-10.1177_0271678X20964248 - Supplemental material for Kinetics and 28-day test–retest repeatability and reproducibility of [11C]UCB-J PET brain imaging [file sj-pdf-10-jcb-10.1177_0271678X20964248.pdf]

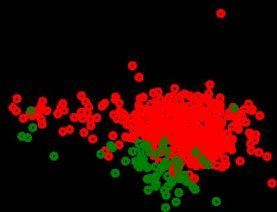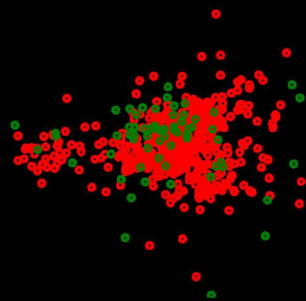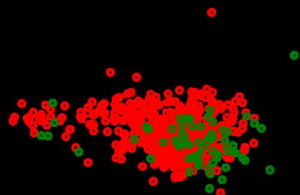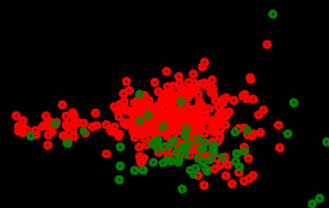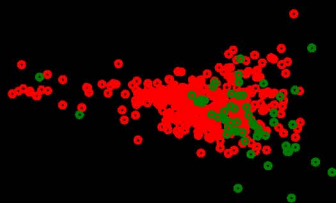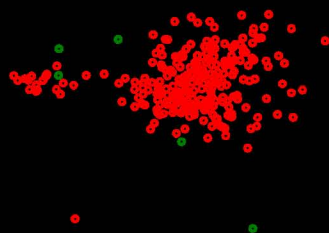

Supplement: sj-pdf-11-jcb-10.1177_0271678X20964248 - Supplemental material for Kinetics and 28-day test–retest repeatability and reproducibility of [11C]UCB-J PET brain imaging [file sj-pdf-11-jcb-10.1177_0271678X20964248.pdf]

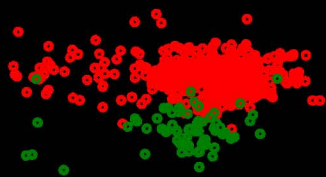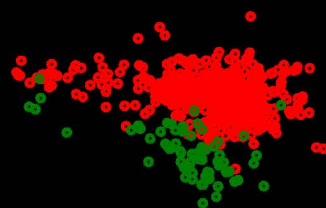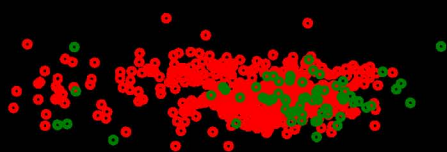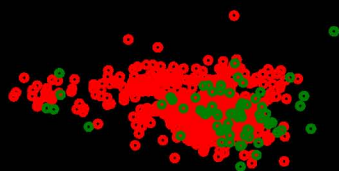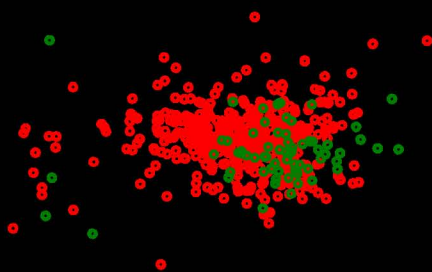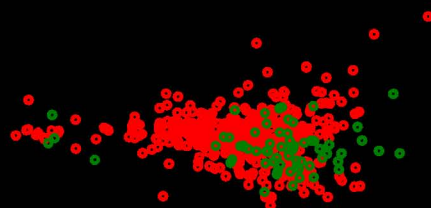

Supplement: sj-pdf-12-jcb-10.1177_0271678X20964248 - Supplemental material for Kinetics and 28-day test–retest repeatability and reproducibility of [11C]UCB-J PET brain imaging [file sj-pdf-12-jcb-10.1177_0271678X20964248.pdf]
