# Supplementary material for: Kinetics and 28-day test–retest repeatability and reproducibility of [11C]UCB-J PET brain imaging
Source: J Cereb Blood Flow Metab. 2020 Oct 8;41(6):1338–50. doi: 10.1177/0271678X20964248 (PMC8138337; doi:10.1177/0271678X20964248)
Supplement: sj-pdf-8-jcb-10.1177_0271678X20964248 - Supplemental material for Kinetics and 28-day test–retest repeatability and reproducibility of [11C]UCB-J PET brain imaging [file sj-pdf-8-jcb-10.1177_0271678X20964248.pdf]

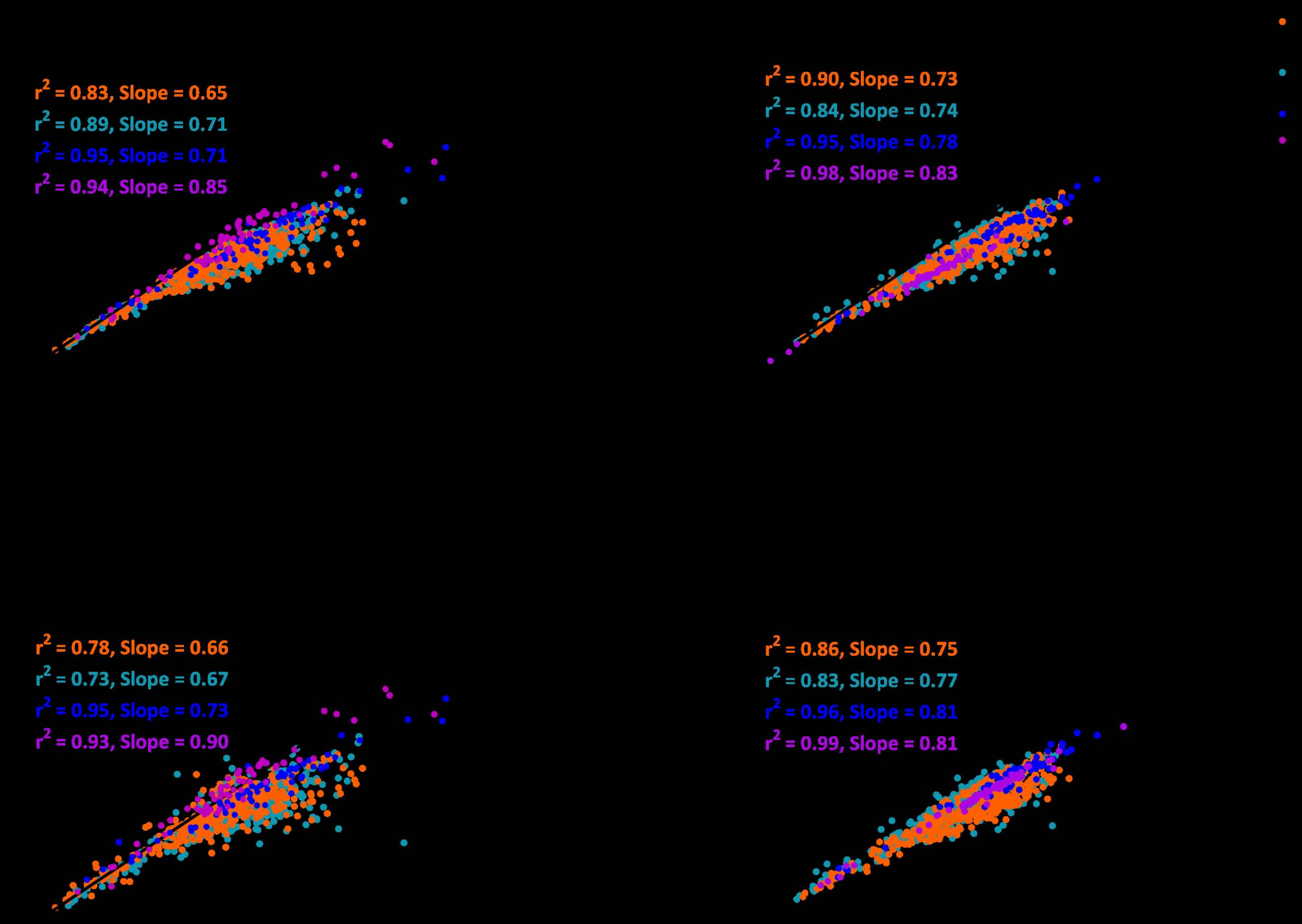
r2 = 0.83, Slope = 0.65
r2 = 0.89, Slope = 0.71
r2 = 0.95, Slope = 0.71
r2 = 0.94, Slope = 0.85
r2 = 0.90, Slope = 0.73
r2 = 0.84, Slope = 0.74
r2 = 0.95, Slope = 0.78
r2 = 0.98, Slope = 0.83
r2 = 0.78, Slope = 0.66
r2 = 0.73, Slope = 0.67
r2 = 0.95, Slope = 0.73
r2 = 0.93, Slope = 0.90
r2 = 0.86, Slope = 0.75
r2 = 0.83, Slope = 0.77
r2 = 0.96, Slope = 0.81
r2 = 0.99, Slope = 0.81
