# Supplementary material for: Kinetics and 28-day test–retest repeatability and reproducibility of [11C]UCB-J PET brain imaging
Source: J Cereb Blood Flow Metab. 2020 Oct 8;41(6):1338–50. doi: 10.1177/0271678X20964248 (PMC8138337; doi:10.1177/0271678X20964248)
Supplement: sj-pdf-13-jcb-10.1177_0271678X20964248 - Supplemental material for Kinetics and 28-day test–retest repeatability and reproducibility of [11C]UCB-J PET brain imaging [file sj-pdf-13-jcb-10.1177_0271678X20964248.pdf]

Supplementary Table 1. Clinical and demographic data.

	HCs (N=9)		AD patients (N=8)	
**Age**	62.2 (5.9)		63.8 (7.8)	
**Males/females (n)**	4/5		5/3	
**MMSE**	29.3 (0.9)		22.5 (3.1)	
**Body length (in cm)**	173 (11.5)		178 (8.3)	
	**Test**	**Retest**	**Test**	**Retest**
**Body weight (in kg)**	76.1 (10.4)	76.2 (10.5)	87.1 (15.3)	87.6 (16.4)
**Injected dose (MBq)**	373 (28)	374 (16)	334 (35)	313 (34)
**Specific activity(GBq/uMo)**	53 (18)	56 (10)	55 (22)	63 (29)

**Note: values indicate mean and SD in brackets.*
