# Supplementary material for: Kinetics and 28-day test–retest repeatability and reproducibility of [11C]UCB-J PET brain imaging
Source: J Cereb Blood Flow Metab. 2020 Oct 8;41(6):1338–50. doi: 10.1177/0271678X20964248 (PMC8138337; doi:10.1177/0271678X20964248)

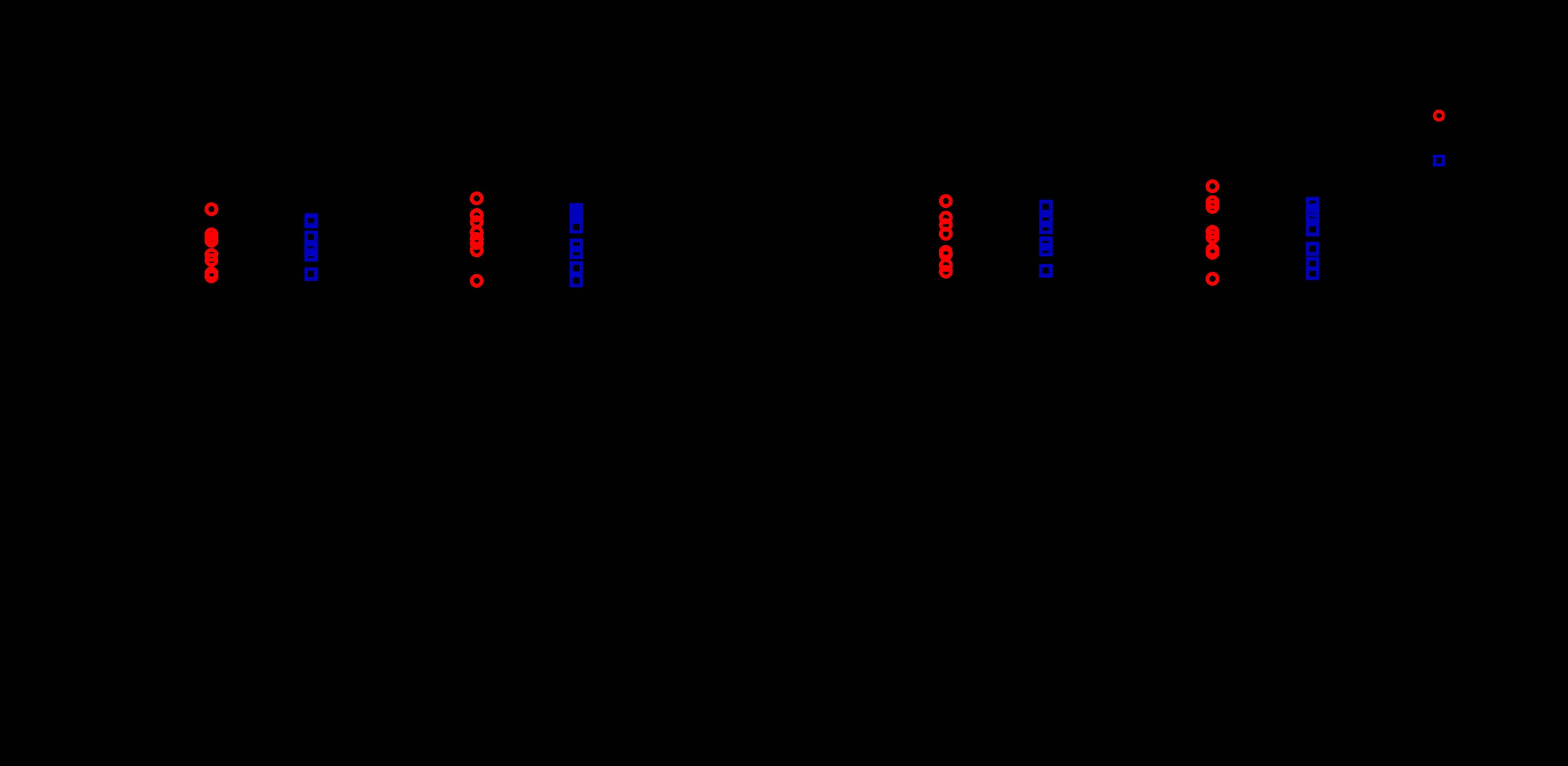

Supplement: sj-pdf-4-jcb-10.1177_0271678X20964248 - Supplemental material for Kinetics and 28-day test–retest repeatability and reproducibility of [11C]UCB-J PET brain imaging [file sj-pdf-4-jcb-10.1177_0271678X20964248.pdf]
